# Green leaves and seeds alcoholic extract controls *Sporobulus indicus* germination in laboratory conditions

**DOI:** 10.1038/s41598-020-58321-y

**Published:** 2020-01-31

**Authors:** Jean Flaviel de Sousa Macêdo, Lylian Souto Ribeiro, Riselane de Lucena Alcântara Bruno, Edna Ursulino Alves, Alberício Pereira de Andrade, Kilson Pinheiro Lopes, Franciscleudo Bezerra da Costa, José Cola Zanuncio, Wellington Souto Ribeiro

**Affiliations:** 1 Departamento de Agroecologia e Agropecuária, Campus II, Universidade Estadual da Paraíba, Sítio Imbaúba s⁄no, 58117-000 Lagoa Seca, Paraíba Brasil; 2 Departamento de Fitotecnia de Ciências Ambientais, Campus II, Universidade Federal da Paraíba, 58397-000 Areia, Paraíba Brasil; 3 Programa de Pós-graduação em Horticultura Tropical, Universidade Federal de Campina Grande, 58.840-000 Pombal, Paraíba Brasil; 4 Departamento de Entomologia/BIOAGRO, Universidade Federal de Viçosa, 36570-900 Viçosa Minas Gerais, Brasil

**Keywords:** Secondary metabolism, Invasive species, Environmental impact

## Abstract

High seed production makes *Sporobolus indicus* var. *pyramidalis* a difficult to control invasive grassland plant. The objective of the present study was to investigate the bioactivity of *Cyperus rotundus*, *Phyllanthus tenellus* and *Ricinus communis* green leaf extracts and of *Carica papaya* seeds on *S. indicus* germination without breaking dormancy, simulating the field conditions. The ethanolic extract bioactivity of *C. rotundus*, *P. tenellus*, *R. communis* green leaves and *C. papaya* seeds, at concentrations of 25, 50 and 75% in *S. indicus* germination was evaluated. Carotenoids, flavonoids, soluble phenolic compounds and total tannins were quantified in the extracts. The chemical component concentrations varied between alcoholic extracts. The *P. tenellus* extracts at all dilutions and those of *R. communi*s and *C. papaya* at 75% completely suppressed *S. indicus* seed germination at five and ten days which can be attributed to their high tannin concentration, total phenolic compounds and flavonoids.

## Introduction

The grass *Sporobolus indicus* var. *pyramidalis* Beauv^1^. is an invasive and aggressive nonnative weed, has become a serious threat in many perennial grass pastures distributed in all tropical regions reducing the quality and production of forage crops^2^.

The *S. indicus* var. *pyramidalis* percentage germination is low (6.7–27%)^2–4^ due to the presence of a hard seed coat^3^. However, the low germination is compensated by high seed production, making it difficult to control. A panicle (30 cm) of *S. indicus* var. *pyramidalis* has around one thousand seeds and one plant can produce more than 200 panicles per year^5^. *S. indicus* spp. produces more than 1,400 seed per panicle and nearly 45,000 seed per plant^3^. Smutgrass seed are thought to remain viable for at least 2 years^6^.

In southern Florida, hexazinone, an expensive herbicide, is the only control option against this plant and it is applied in pastures when infestations of this grass reach 30%^5,7,8^. In Brazil, *S. indicus* var. *pyramidalis* is controlled with glyphosate (360 g L^−1^) or manually, in small areas when at low densities or in organic and agroecological production systems. In these systems, the plants are ripped, bagged and burned far from the pasture, but its seed reserve in the soil is large. Chemical products to control of *S. indicus* var. *pyramidali*s, are expensive or dangerous and its intensive use in integrated systems is a problem. This makes it necessary to develop strategies to manage this plant, including products based on plant extracts, mainly for organic and agroecological production^9^. In addition, modern agriculture seeks natural organic methods to reduce the extensive and intensive application of chemicals, that impact the environment, public health, and the cost of agricultural production^10,11^.

Allelochemicals, produced during secondary plant metabolism, may reduce the growth, survival and reproduction of invading species^12,13^. Phenolic compounds are allelochemicals deriveted the shikimic and acetic acid (polyketide) metabolic pathways in plants^14^. *Cyperus rotundus* L. (Cyperaceae), *Phyllanthus tenellus* Roxb. (Phyllanthaceae), *Ricinus communis* L. (Euphorbiaceae) and *Carica papaya* L. (Caricaceae) seeds have toxicological properties. Gallic acid, chlorogenic acid, 3,4-dihydroxybenzaldehyde, p-hydroxybenzoic acid, catechol, tannic acid, ricinine are some of the allelochemical phenolic compounds found in these species^15^. But the allelopathic potential of these plants on seeds weeds needs to be better studied^10^. Aqueous extracts have been studied^16–18^, but many non-polar bioactive substances cannot be dissolved by water at room temperature, unlike organic solvents^13^. Polar solvents such as methanol, ethanol, acetone, or acetonitrile give much high extraction efficiencies^14^.

Phenolic compounds, originated to protect plants from oxidative damage, are also involved in plant allelopathy inducing changes in membrane permeability, inhibition of nutrient uptake, cell division, stretching and submicroscopic structure, altering enzyme activity, respiration, and synthesis of hormones and proteins^14^. Studies on the performance of phenolic compounds such as allelopaths can provide data to development sustainable methods of agriculture, forestry, natural resources and conservation of the environment.

The objective of the present study was to evaluate the bioactivity of alcoholic extracts of *C. rotundus*, *P. tenellus*, *R. communis* green leaves and *C. papaya* seeds with on the *S. indicus* var. *pyramidalis* germination without breaking dormancy, simulating the field conditions.

## Results

The concentration of the chemical components varied between and among the alcohol extracts of *C. rotundus*, *P. tenellus* and *R. communis* green leaves and that of *C. papaya* seeds. Total flavonoids ranged from 0.32 (*P. tenellus* to 25%) to 10.00 mg 100 g^−1^ (*C. rotundus* to 75%). Total tannins from 0.24 (*C. papaya* 25%) to 16.32 mg catechin g^−1^ (*P. tenellus* 75%). Soluble phenolic compounds from 0.89 (25% *P. tenellus*) to 754.23 mg kg^−1^ (*C. papaya* 75%). Total carotenoids from 0.09 (*R. communis* at 25%) to 7.00 mg 100 g^−1^ (*C. papaya* at 75%) (Table 1). *P. tenellus* extracts at all dilutions and those of *R. communis* at 75% and *C. papaya* extracts completely suppressed *S. indicus* seed germination up to 30 days after aplication (Table 2).

**Table 1:** Total flavonoids (mg 100 g^−1^) (TF), total tannins (mg of catechin g^−1^) (TT), soluble phenolic compounds (mg kg^−1^) (SPC) and total carotenoids (TC) in alcoholic plant extracts with 25, 50 and 75% dilution. Extracts concentration (C%).

Species	C%	TF	TT	SPC	TC
*Phyllanthus tenellus*	25	0,32 ± 0,03	4,87 ± 0,34	0,89 ± 0,09	—
	50	0,97 ± 0,03	7,32 ± 0,56	1,25 ± 0,11	—
	75	1,47 ± 0,02	16,32 ± 0,58	3,01 ± 0,13	—
*Cyperus rotundus*	25	4,76 ± 0,40	4,08 ± 0,20	79,65 ± 5,34	—
	50	6,98 ± 0,67	6,97 ± 0,30	86,89 ± 4,98	—
	75	10,00 ± 0,73	8,13 ± 0,61	187,17 ± 12,78	—
*Carica papaya*	25	0,42 ± 0,02	0,24 ± 0,03	215,87 ± 32,87	2,50 ± 0,13
	50	0,99 ± 0,03	0,31 ± 0,10	327,77 ± 45,87	3,98 ± 0,43
	75	1,76 ± 0,02	0,47 ± 0,10	754,23 ± 54,44	7,00 ± 1,02
*Ricinus communis*	25	—	2,13 ± 0,10	5,78 ± 0,23	0,09 ± 0,03
	50	—	4,56 ± 0,10	11,76 ± 0,90	0,13 ± 0,01
	75	—	7,89 ± 0,10	20,17 ± 1,03	0,45 ± 0,01

**Table 2:** Germination percentage of *Sporobolus indicus* var. *pyramidalis* seeds treated with plant extracts. Extracts concentration (C%).

Species	C%	Germination (%)					
		5th day	10th day	15th day	20th day	25th day	30th day
*Phyllanthus tenellus*	0	0 ± 0.0a	21 ± 1.4a	20 ± 0.9a	20 ± 1.6a	19 ± 0.7a	21 ± 1.1a
	25	0 ± 0.0a	0 ± 0.0b	0 ± 0.0b	0 ± 0.0b	0 ± 0.0b	0 ± 0.0b
	50	0 ± 0.0a	0 ± 0.0b	0 ± 0.0b	0 ± 0.0b	0 ± 0.0b	0 ± 0.0b
	75	0 ± 0.0a	0 ± 0.0b	0 ± 0.0b	0 ± 0.0b	0 ± 0.0b	0 ± 0.0b
*Cyperus rotundus*	0	0 ± 0.0a	19 ± 1.9a	19 ± 1.5a	18 ± 0.7a	17 ± 1.1a	18 ± 0.9a
	25	0 ± 0.0a	14 ± 2.4b	14 ± 2.5b	15 ± 2.0b	14 ± 1.0b	14 ± 1.8b
	50	0 ± 0.0a	14 ± 3.0b	15 ± 2.1b	15 ± 1.5b	13 ± 2.0b	15 ± 1.5b
	75	0 ± 0.0a	1 ± 0.4c	1 ± 2.6c	1 ± 2.5c	1 ± 2.0c	1 ± 1.5c
*Ricinus communis*	0	0 ± 0.0a	20 ± 0.7a	21 ± 1.2a	21 ± 0.9a	23 ± 1.6a	21 ± 0.9a
	25	0 ± 0.0a	23 ± 0.3a	20 ± 1.5a	21 ± 0.9a	20 ± 1.7a	21 ± 1.1a
	50	0 ± 0.0a	7 ± 0.9b	6 ± 1.3b	7 ± 0.9b	5 ± 0.9b	7 ± 1.0b
	75	0 ± 0.0a	0 ± 0.0c	0 ± 0.0c	0 ± 0.0c	0 ± 0.0c	0 ± 0.0c
*Carica papaya*	0	0 ± 0.0a	19 ± 1.1a	19 ± 1.1a	20 ± 1.7a	19 ± 1.2a	20 ± 0.7a
	25	0 ± 0.0a	15 ± 2.7b	15 ± 1.4b	15 ± 2.9b	15 ± 1.7b	15 ± 2.0b
	50	0 ± 0.0a	12 ± 1.3b	13 ± 1.7b	14 ± 3.3b	12 ± 1.9b	15 ± 2.7b
	75	0 ± 0.0a	0 ± 0.0c	0 ± 2.7c	0 ± 0.0c	0 ± 2.7c	0 ± 0.0c

^*^Means followed by the same letter per column do not differ (P < 0.05 Kruskall-Wallis test) by Mann–Whitney *U*-test.

## Discussion

The variation in the concentration of chemical components between the alcoholic extracts confirms their wide occurrence and diversity in plants^19,20^ as reported for *Artemisia campestres* L. (Asteraceae), *A. Herba halba* L. (Asteraceae), *A. arboresens* L. (Asteraceae), *A. arvensis* L. (Asteraceae), *Juniperus oxycedrus* L. (Cupressaceae), *Globularia alypum* L. (Globulariaceae), *Oudneya africana* R. Br. (Brassicaceae), *Monuta Rout*e L. (Rutaceae), *Thapsia garganica* L. (Apiaceae), *Thymelaea hirsuta* L. (Thymelaeaceae) and *Teucrium polium* L. (Lamiaceae)^21^ and thirty-two other herbs^22^. This makes plants from different habitats in Sardinia, Italy^23^ of the same species growing in different conditions^24^ have chemical composition variation as reported for *Myrtus communis* L. (Myrtaceae). The variation in the concentration of the chemical components between the alcoholic extracts is due to their proportions in the solvent/solute (dilution) which determines the effectiveness of the plant extracts and the isolated compounds^25^. In addition to dilution, the solvent may also alter the chemical composition of the extracts, as reported for *Origanum vulgare* L. (Lamiaceae)^26^, *Anthocleista grandiflora* Gilg. (Gentianaceae) and *Combretum erythrophyllum* Burch. (Combretaceae) in wich compound quantity and diversity varied according to the extractors and their concentration^25^.

Flavonoids, a group of phenolic compounds resulting from secondary metabolism, are widely found in plants^27^ and their higher amount in the 75% *C. rotundus* extract agrees with that reported for the rhizome extract of this plant^28–31^. However, abiotic and biotic stress^26^ and changes in seasonal dynamics can affect compound content and when it is higher in the plant it will also be in the extract, as reported for *Dryopteris erythrosora* (DC Eaton) Kuntze (Dryopteridaceae) with the transport of flavonoids from the leaves to the stem in the growing season, comprising summer (26.9 °C) and early autumn (16.9 °C) in Shanghai, China^32^. The highest total tannin levels (another phenolic compound group) from the *P. tenellus* extract could be a response to the stressful environment in which this invasive species was collected^33^, area with stones, few soil and water deficit, in the microregion of Campina Grande, Paraíba, Brazil, with few soil and water deficits. The tannin accumulation, in this case, has an antioxidative function^34^ and agrees with the phytochemical profile of the methanolic solution (80%) of the whole *P. tenellus* plant^35^. The highest phenolic compound content and total carotenoids in the *C. papaya* L. seed extract is due to its function in sanity and resistance to pests and diseases, as a strategy for seed survival^36^, mainly against oxidative stress^37^. These compunds act in response to environmental stress conditions protecting against injuries, as reported in the identification of the phenolic profile of papaya fruits^36,38^. Secondary products of metabolism such as flavonoids, tannins, phenolic compounds and carotenoids^39^, may act in inhibiting germination^40^ reducing tissue growth or causing death by increasing cell membrane permeability, as reported for *Cucumis sativus* L. (Cucurbitaceae)^41^, *Lactuca sativ*a L. (Asteraceae)^14^, *Phaseolus vulgaris* L. (Fabaceae)^42^ resulting in the inhibition of radicular elongation and ultra structural changes and cell division.

The suppression of *S. indicus* germination by *C. papay*a, *P. tenellus* and *R. communis* extracts may be due to their high tannin concentration and total phenolic compounds (derived from the acetate and shikimic acid route or their combination)^39^. These compounds bind strongly to proteins by hydrogen bonds and hydrophobic interaction, deactivating them and blocking germination metabolism^43,44^ or preventing the access of free oxygen to the embryo and the release of carbon dioxide^45^. This was reported for *Sorghum bicolor* L. Moench. (Poaceae) which tannin content was correlated with its germination. The highest flavonoid concentration in *C. rotundus*, *R. communi*s and *C. papaya* extracts at 75%, also explains the allelopathic effect on *S. indicus* germination. In addition, the flavonoids are compounds with high antioxidant power^46^ suppressing germination by inhibiting the indole-acidase oxidase (IAA oxidase), gibberellic acid (GA_3_) and indo-3-acetic acid (IAA)^47^. The allelopathic effect of *Dittrichia viscosa* (L.) W. Greuter extracts was attributed to flavonoids^48^ and, even at low concentrations (0.1–1.0%), those of *Ocimum gratissimum* L. (Lamiaceae) inhibited the germination and growth of corn and beans^49^. The suppressive germination effect by the *C. papaya* extract at 75% may also be due to caricacin^50^, which suppresses cell division and phytohormone production and increases the permeability of membranes, inhibiting germination^43,51^. The absence of toxicity of the *R. communis* extract at 25 and 50% can be explained by their adsorption by allelopathic active compounds such as sugars and other *S. indicus* seed carbohydrates, whereas this was not sufficient at 75% concentration due to the high concentration. High sugar concentrations as well as of other carbohydrates, such as glucose and fructose, maltose, sucrose, raffinose, myo-inositol and galactinol have been reported for *Poa annua* L.^52^, *Melinis minutiflora* P. Beauv. (Poaceae), *Echinolaena inflexa* Poir. (Poaceae), and *Lolium multiflorum* L. (Poaceae)^53^ from the same *S. indicus* family. Ricin, a highly toxic *R. communis* heterodimeric protein is composed of polypeptide chains with an affinity for cell surface carbohydrates^54–57^ becomes inert when adsorbed by them thereby not influencing germination^18,58,59^. Lectin, a N-acetylgalactosamine present in seeds, including those of the Poaceae family^60^ is another protein class with reversible carbohydrate binding capacity that can adsorb ricin and other allelopathic compounds, deactivating the *R. communis* extract atby inhibiting the germination of the invasive plant *S. indicus* var. *pyramidalis*.

## Conclusion

*Phyllanthus tenellus* alcohol extracts at all *R. communis* concentrations and *C. papaya*, at 75%, suppressed the germination of *S. indicus* var. *pyramidalis*. These extracts have the potential to manage this plant in organic and agroecological production systems.

## Material and Methods

### Raw material, preparation and characterization of extracts

Extracts were obtained from *C. rotundus*, *P. tenellus* and *R. communis* green leaves and *C. papaya* seeds by immersion in 70% ethyl alcohol for seven days^14^. The alcohol was extracted at 250 °C and the extract filtered and diluted in distilled water to obtain the concentrations of 25, 50 and 75% and their effects were compared with distilled water (control). The chemical composition of extracts at all concentrations was characterized.

### Soluble phenolic compounds

The extracts were prepared by adding 10 mL of methanol: acetic: water solution (50:3.7:46.3) to 10 mg of extract, sonicated for 15 min and centrifuged (NT810 model, Nova Técnica Ind. Com. Equipamentos para Laboratório LTDA, Brazil) at 16,000 rpm min^−1^ for 15 min. An aliquot of the extract (0.2 mL) was withdrawn and 1:10 (v/v) Folin-Ciocalte: water solution added. The final solution was incubated for 10 min at room temperature^14^. A total of 0.8 ml of sodium carbonate (7.5%) was added to the resulting solution, which was mixed and incubated for 30 min at room temperature. Soluble phenolic compound concentrations were determined using UV-Vis spectrophotometer (4001/4 model, Spectronic® 20 GenesysTM, USA) at 473 nm with gallic acid as standard.

### Total flavonoids

Flavonoids were extracted with ethyl alcohol solution (95%) - HCl (1.5 N) at the ratio 85:15^15^. An aliquot of 10 mL of the extract solution was added to 1.0 g of the alcoholic extract. The samples were vortexed for 2 min and the contents packed in amber flasks for 24 h at 4 °C. After 24 h, the material was centrifuged at 3,500 rpm (2,380 × *g*) for 10 min and the supernatant removed. The volume was completed to 10 ml and readings were performed using a UV-Vis spectrophotometer (4001/4 model, Spectronic^®^ 20 GenesysTM, USA) at 374 nm with the results expressed in mg 100 g^−1^.

### Total carotenoids

Total carotenoids were extracted in a steel vessel with an aliquot of 2.0 g of alcoholic extract, 6.0 mL of isopropyl alcohol and 2.0 mL of hexane stirred for 2 min^61^. The contents were transferred to an amber 125 mL separatory funnel, making up the volume with water. After 30 min resting, the material was washed, repeating the operation three times. The contents were filtered with powdered cotton wool with anhydrous sodium sulfate into a 10 mL volumetric flask wrapped with aluminum with 2.0 mL of acetone and the volume made up with hexane. The readings were performed in a UV-Vis spectrophotometer at 450 nm and the results expressed in mg 100 g^−1^.

### Total tannins

The samples of alcoholic extracts were allowed to stand for 1 h in 40 mL of 50% methyl alcohol, centrifuged at 15,000 rpm for 15 min and the supernatant transferred to a 100 mL volumetric flask. A 70% acetone solution was added to the precipitate, which was kept standing for a further 1 h. The mixture was again centrifuged at 15,000 rpm for 15 min and the supernatant discarded. The precipitate was placed in a thermostatic bath at 100 °C for 3 h, cooled in an ice bath, filtered into a 50 ml volumetric flask and the volume filled with the extractive solution. The readings were made in 6 mL aliquots of butanol: HCl and 0.2 mL of 2 N:FeNH_4_(SO_4_).12H_2_O per test tube. After stirring, these tubes were placed in a thermostatic bath at 100 °C for 50 min and cooled in an ice bath. The reading was performed in a UV-Vis spectrophotometer at 550 nm and the results expressed in mg of catechin g^−1^.

### Panicles collection

Panicles without evidence of herbivory and fungi and with mature seeds were collected from plants distributed in ten (10) farms with pastures infested by *S. indicus* var. *pyramidalis* in the state of Paraíba, northeastern Brazil. Mature seeds were randomly selected and naturally dried. The viability test was performed in duplicate in batches of 100 seeds of each property. The viability test was performed for 30 days^62^. However, seeds that did not germinate within 10 days were rotting^4^.

### Bioassay

*S. indicus* germination was evaluated in triplicate with 100 seeds every 10 days in a germination box (Gerbox^®^) (11 × 11 × 3.5 cm) with two germination paper (Germitest^®^) moistened with 18 ml of the different extracts and distilled water in the control. Seed dormancy were not broken to simulate field conditions. The germination assays were done in a germination chamber at 20 °C with 14 h light per daily. Germination was evaluated daily by 10 days^4^. Seeds with radicle protrusion were considered germinated. The percentage of germination was obtained with the formula: % G = (N/A)*100, where: N = total number of seeds germinated; A = total number of seeds placed to germinate^63–67^.

### Experimental design and statistical analysis

The experimental design was completely randomized with three replicates of 100 seeds. The germination rates was compared across independent samples by using non-parametric Kruskal Wallis H test. Further, Mann Whitney *U* test was used to compare the two germination rates.
